# Gaming Activity and Possible Changes in Gaming Behavior Among Young People During the COVID-19 Pandemic: Cross-sectional Online Survey Study

**DOI:** 10.2196/33059

**Published:** 2022-01-25

**Authors:** Emma Claesdotter-Knutsson, Frida André, Anders Håkansson

**Affiliations:** 1 Department of Clinical Sciences Lund Faculty of Medicine Lund University Lund Sweden; 2 Gambling Disorder Unit, Malmö Addiction Centre Department of Clinical Sciences Lund, Faculty of Medicine Lund University Lund Sweden

**Keywords:** COVID-19 pandemic, gaming, screen time, psychological distress

## Abstract

**Background:**

Young people’s daily lives and social interactions changed remarkably during the COVID-19 pandemic as schools and cinemas closed, leisure activities were cancelled, and gatherings were regulated. Questions have been raised by the media, schools, policy makers, and research communities about the effect on young people’s online behaviors.

**Objective:**

This cross-sectional study aimed to study self-reported changes in gaming, focusing on a younger section of the population during the COVID-19 pandemic in Sweden. We also wanted to look at potential risk factors behind problematic gaming during the pandemic, including gaming patterns, gambling behavior, psychological distress, certain sociodemographic characteristics, health factors, and school situation.

**Methods:**

This was an anonymous online survey study of web panel participants in Sweden (n=1501) to study changes in gaming behaviors during the COVID-19 pandemic. Self-reported increases in gaming were analyzed in logistic regression analyses against sociodemographic and health factors.

**Results:**

Within the study population that reported changes in gaming activity, we found significant differences in age, employment status, disposable income, whether they ever played on loot boxes, time spent at home, school attendance, psychological distress, and gambling and gaming problems, as well as significant differences in changes in alcohol consumption and exercise habits. When examining the 16–24-year-old age group who reported changes in gaming activity, we found significant differences within the group in disposable income, time at home, and school attendance. When examining the 25–39-year-old age group who reported changes in gaming activity, we found significant differences within the group in employment status, disposable income, time spent at home, whether the respondents were studying, school attendance level, psychological distress, and gaming problems, as well as significant differences in changes in alcohol consumption and exercise habits. Psychological distress (all age groups analyzed together; 25–39-year-old age group), drinking less alcohol (all age groups analyzed together), spending more time at home (all age groups analyzed together), gaming problems, and exercising less (25–39-year-old age group) were positively correlated with a self-reported increase in gaming activity. Being employed (25–39-year-old age group) and being over 40 years of age (all age groups analyzed together) were negatively correlated with increased gaming. We found no significant correlations in the 16–24-year-old age group.

**Conclusions:**

Those who reported increased gaming during the COVID-19 pandemic were more likely to be 16 years to 39 years old. In the age group of 25 years to 39 years old, the increase was associated with psychological distress, reporting less exercise, and being unemployed. COVID-19 may present as a risk factor of increased online gaming in a small but vulnerable group. More research and preferably longitudinal studies are needed in the field of gaming and effects of the COVID-19 pandemic.

## Introduction

The index case of the now widespread COVID-19 pandemic caused by the SARS-CoV-2 virus originated in Wuhan, the capital of Hubei Province, China, on December 8, 2019 [1]. On January 31, 2020, the first confirmed case of SARS-CoV-2 viral infection was recorded in Sweden. In subsequent months, COVID-19 reached most countries in Europe [2], and on March 11, 2020, the World Health Organization (WHO) declared the outbreak a pandemic.

As well as physiological harm, the COVID-19 pandemic has also had an enormous effect on people’s mental health [3,4]. Research has confirmed that rates of depression, addiction, anxiety, and other psychiatric disorders rose during the pandemic [5-10]. Research has shown an increase in screen time and the consumption of digital entertainment during the pandemic, particularly online gaming and related activities such as video game streaming [4,9,11-13]. This phenomenon has been seen worldwide. In India, for example, WinZO Games reported 300% greater user engagement, 30% higher traffic in online mobile gaming, and 35% higher usage in multiplayer modes [14], and another Indian mobile-based online gaming platform reported an almost 200% increase in the user base during the pandemic, with 75,000 new users [15]. A 70% increase in Fortnite gaming has been seen in Italy [16]. In the United States, one of the largest telecom providers reported a 75% rise in online activity [17]. In 2020, the world’s largest video game digital distribution service, Steam, reported 20 million active users, an all-time high [18]. The WHO has supported gaming as an activity that promotes social distancing and reduces the loneliness that might follow, including the gaming industry’s media campaign #PlayApartTogether, which mixes guidelines to prevent the spread of COVID-19 with messages encouraging online gaming [19-22]. It has been suggested that online gaming could be psychosocially beneficial to young people—cognitively, motivationally, emotionally, and socially [23,24]—and especially so in the COVID-19 pandemic, when recommendations and regulations to stop the spread of the virus have affected everyone’s lives. Gaming has been shown to reduce loneliness [25,26] and, even though concerns have been raised about its addictive potential, research has shown that frequent gaming does not have to be problematic [27] and most gamers’ gaming habits are changeable and not fixed [28,29]. It has also been suggested that gaming could work as a coping mechanism against stress [30,31]. However, excessive screen time has been shown to be associated with a range of negative mental health outcomes, including anxiety and depression, in adolescents considered particularly vulnerable to a problematic use of online media [32-34]. Softening its excessively positive initial message about gaming, the WHO’s mental health information (#HealthyAtHome—Mental Health) recommended a *balance* between time spent on screens—and gaming in particular—and offline activities [22].

Governmental virus protection strategies have differed around the world from recommendations to more mandatory interventions [35]. Sweden’s approach to prevent virus spread has not included lockdown or stay-at-home orders but rather recommendations. Most workplaces encouraged their workers to work from home, and regulations in opening hours have been in effect for places such as restaurants and shopping malls, leading people in general to spend a lot more time inside with possible access to screens.

Young people’s daily lives and social interactions have changed remarkably in the COVID-19 pandemic because of school and cinema closures, the cancellation of leisure activities, and restrictions on gatherings. Without the daily routine of going to school, young people had unlimited opportunities to play video games, making them vulnerable to developing problems related to excessive gaming.

Internet gaming disorder (IGD), it should be noted, is included in the 11th revision of the International Classification of Diseases (ICD 11), defined as a gaming behavior of sufficient severity to result in significant impairment in areas of function [36]. However, the *Diagnostic and Statistical Manual of Mental Disorders* (DSM-5) has described IGD as “necessitating further clinical experience and research before inclusion as a formal disorder” [37].

With regard to gambling habits, there has also been considerable research on the impact of the COVID-19 pandemic [38]. Our research in this area has shown that the increase in gambling in the general population in Sweden is limited, but the group who does report increased gambling activity also reports psychosocial problems [38-41]. Researchers have raised the concern that more time spent at home *could* increase gaming and its possible negative side effects, but there is very little research on the *actual* situation [18]. We thus set out to see if gaming habits changed during the COVID-19 pandemic in the same way as gambling habits.

The aim of this cross-sectional study was thus to study self-reported changes in gaming during the COVID-19 pandemic, focusing on a younger section of the Swedish population. We also considered the potential risk factors for problematic gaming during the pandemic, including gaming patterns, gambling behavior, psychological distress, certain sociodemographic characteristics, health factors, and school situation.

## Methods

### Setting

The first wave of the COVID-19 pandemic in Sweden was in the spring of 2020. In the summer of 2020, virus transmission decreased, and the second wave started in the autumn of 2020. After only a partial decrease in virus transmission in the winter of 2020-2021, early 2021 saw a third wave of virus transmission and an increased hospital-admitted disease burden in Sweden [42]. This study was cross-sectional and based on a self-report online survey study carried out in Sweden in March 2021 during the third wave of the ongoing COVID-19 pandemic in the country. At the time of the study, secondary schools in Sweden had started to open up for on-site teaching, but the national COVID-19 strategies regarding leisure activities and restrictions on gatherings of more than 8 people were still in effect.

### Participants and Procedures

We used the market survey company Ipsos and their online web survey panel. Ipsos is a market survey company that has broad experience in conducting survey studies in the area of addictive disorders. Ipsos abides by the International Chamber of Commerce (ICC)/ESOMAR Code. The Ipsos web panel has previously been used for online surveys in the course of our research [43]. In this study, we invited respondents from the general population, aged 16 years and older. Participants for the Ipsos web panel were invited with the information that the survey would address “computer gaming, gambling for money and other behavioral patterns in Sweden during COVID-19—association with mental health, social situation and attitudes to the pandemic.” The survey was made accessible only when the respondent provided electronic informed consent. Participants on the Ipsos web panel enroll voluntarily to take market surveys, political opinion polls, and similar surveys, for which they earn points they can redeem as goods or services. Most surveys are worth a point and each point is worth approximately €1 (US $1.14). The survey is sent out to different age groups of web panel participants until a gender and age distribution close to that of the general population is achieved. In this study, invitations were sent until some 1500 complete answers were obtained. In addition, in this study, the final distribution of age groups, gender, and geographical location (regions) was compared by Ipsos with those of the general population, such that the data set was weighted according to a summarized weighting score derived from these 3 variables. When the survey was halted, the final sample consisted of 1501 individuals. The study was carried out from March 19, 2021 through March 29, 2021. The study was reviewed and approved by the Swedish Ethical Review Board (File: 2021/00369).

### Measures

Basic sociodemographic variables comprised gender (female or male), age (divided into 2 age groups: 16-24 years; ≥25 years), monthly income (divided into 3 groups: SEK 10,000-20,000 [US $1107.48-$2214.97]; SEK 20,000–40,000 [US $2214.97-$4429.94]; SEK ≥40,000 [US ≥$4429.94]), level of education (university, secondary school [age 16-19 years], primary school [age 6-16 years], other), employment status (studying, employed, unemployed, retired, other). The questionnaire began with questions about changes in the respondent’s personal behavior during the COVID-19 pandemic (“since these changes in Sweden started”); whether they, during this period, had spent more or less time at home (much more, slightly more, unchanged, or less time at home); and whether they had consumed more or less alcohol (more, less, unchanged, or don’t drink at all). Thereafter, questions were asked regarding schooling situation—whether the respondents attended school of any kind including university (yes or no) and whether their level of achievement had been affected by the remote learning situation during the pandemic (for the better, for the worse, or not affected). Finally, the participants were asked if their school attendance had been affected by the remote learning situation during the COVID-19 pandemic (less absence, more absence, or not affected). The final questions were about gaming, specifically whether their personal gaming habits had changed, excluding games for money (more, no change, less, I do not engage in gaming). We also asked about loot boxes: whether in the last 12 months they had engaged in gaming involving currency inside a video game, with the purpose to either gain money or advantages in the video game (yes, no).

The Game Addiction Scale for Adolescents (GASA) is one of the most frequently used questionnaires for gaming addiction [44-47]. The scale was theoretically based on the DSM-5 criteria for pathological gambling [47]. Each question covers 1 criterion, answered on a 5-point continuum scale: 1 (never), 2 (rarely), 3 (sometimes), 4 (often), 5 (very often). It should, according to the developer, be accounted as endorsed when rated 3 or higher. The DSM-5 requires half (or more) of their criteria to be met, while scholars within the field of gaming suggest a ranking of the criteria: the “core approach.” The criteria tolerance, mood modification, and cognitive salience are said to be associated with engagement rather than addiction, while the contrary applies for the criteria withdrawal, relapse, conflict, and problems [47-49]. In order to distinguish between levels of severity within the group of gamers, the core approach was applied, whereby the individuals who met all the core criteria (relapse, withdrawal, conflict, and problems) constituted the group *addicted gamers*. The respondents who endorsed 2 or 3 of the core criteria but none of the peripheral criteria (salience, tolerance, mood modification) were grouped as *problem gamers*, and those that endorsed all 3 of the peripheral criteria but not more than one of the core criteria were grouped as *engaged gamers* [46,50]. The remaining respondents comprised the group “*no* *problem” gamers*, as individuals below the cut-off for engaged gaming.

Since both the problem gamers and the addicted gamers were assumed to be associated with more severe gaming behavior as well as more negative outcomes [46,51], these 2 groups also constituted one combined group (2-4 endorsed core criteria): *addicted/problem gamers*.

Psychological distress was measured using the Kessler-6 scale [52]. This scale measures symptoms of depression and anxiety perceived during the past 6 months. The Kessler scores (0-4 for each question) were summed, and a total score of 5 or more was classified as psychological distress [52].

The level of potential gambling problems was measured with the 9-item Problem Gambling Severity Index (PGS-I) [53], where each of the statements addresses the preceding 12 months. For the PGS-I scale, the scores of 0-3 for each question were summed: A score of 0 indicated no problem with gambling; a score of 1-2 indicated a low risk of gambling problems; 3-7 indicated a moderate risk of gambling problems; and 8 or more indicated gambling problems.

### Statistical Analysis

Weighting adjustments were applied to compensate for nonresponse and noncoverage and to make the sample estimates conform to external values. The reporting of prevalence measures and group-wise comparisons related to the weighted data and statistical tests were applied using the Chi-square test, while binary logistic regression analyses were carried out using the unweighted data. Binary logistic regression analyses were carried out with increased changes in gaming behavior (yes/no) as the dependent variable in order to study potential independent variables associated with the outcome. For all models, odds ratios (ORs) with 95% CIs are presented. For all statistical analyses, SPSS version 25.0 (IBM Corp, Armonk, NY) was used [54].

## Results

### Descriptive Data on Changes in Gaming Behavior by Demographic and Socioeconomic Factors (Weighted Data)

#### Overview

The descriptive data for the sample are shown in Table 1. The questionnaire was answered by 1501 participants, of whom 37.9% (569/1501) answered they had never played video games, neither now nor before the pandemic, and were therefore removed from further analysis. The remaining study population (932/1501) was used for further analysis. We chose to study the younger section of the population. Of the population who did game, 16.4% (153/932) were in the 16–24-year-old age group, and 30.8% (287/932) were in the 25–39-year-old age group. Gender was equally distributed in both age groups. We then looked at the various demographic and socioeconomic factors described in the previous sections and their relationships with self-reported changes in gaming activity (increased, decreased, unchanged).

**Table 1:** Survey respondents and the final study populations by age group.

Characteristics		Total sample (n=1501), n (%)	Age groups	
			16-24 years (n=190), n (%)	25-39 years (n=392), n (%)
Never gamed		569 (37.9)	37 (19.5)	105 (26.8)
Study population		932 (62.1)	153 (80.5)	287 (73.2)
**Gender**				
	Female	452 (48.5)	68 (44.4)	152 (53.0)
	Male	480 (51.5)	85 (55.6)	135 (47.0)

#### All Age Groups

We found significant differences according to age group (*P*<.001), employment status (*P*<.001), disposable income (*P*<.001), whether respondents ever played on loot boxes (*P*=.002), time spent at home (*P*<.001), changes in alcohol consumption (*P*<.001), changes in exercise habits (*P*<.001), whether the respondents were studying (*P*<.001), school attendance (*P*<.001), Kessler score (*P*<.001), PGS-I score (*P*<.001), and GASA score (*P*<.001; Table 2).

**Table 2:** Gaming behavior by demographic characteristics among all participants.

Characteristics			Gaming behavior, n (%)							*P* value			Participants, n (%)
			Increased		Unchanged		Decreased						
Total sample			357 (38.3)		541 (58.0)		34 (3.6)		N/A^a^			932 (100)	
**Gender**													
	Female	179 (39.6)		259 (57.3)		14 (3.1)		.55			452 (48.5)		
	Male	178 (37.1)		282 (58.8)		20 (4.2)					480 (51.5)		
**Age group (years)**													
	16-24	87 (56.9)		59 (38.6)		7 (4.6)		<.001			153 (16.4)		
	25-39	146 (50.9)		126 (43.9)		15 (5.2)					287 (30.8)		
	40-59	77 (25.8)		213 (71.2)		9 (3.0)					299 (32.1)		
	≥60	47 (24.4)		143 (74.1)		3 (1.6)					193 (20.7)		
**Employment status**													
	Studying	93 (61.2)		52 (34.2)		7 (4.6)		<.001			152 (16.3)		
	Employed	174 (33.4)		325 (62.4)		22 (4.2)					521 (55.9)		
	Unemployed	34 (54.0)		28 (44.4)		1 (1.6)					63 (6.8)		
	Retired	48 (27.7)		122 (70.5)		3 (1.7)					173 (18.6)		
	Other	8 (34.8)		14 (60.9)		1 (4.3)					23 (2.5)		
**Level of education**													
	Primary school	37 (43.5)		47 (55.3)		1 (1.2)		.60			85 (9.1)		
	Secondary school	135 (36.9)		214 (58.5)		17 (4.6)					366 (39.3)		
	University	173 (38.4)		261 (58.0)		16 (3.6)					450 (48.3)		
	Other	12 (38.7)		19 (61.3)		0 (0.0)					31 (3.3)		
**Disposable income (SEK^b^)**													
	<20,000	150 (48.7)		146 (47.4)		12 (3.9)		<.001			308 (33.0)		
	20,000-40,000	157 (34.5)		284 (62.4)		14 (3.1)					455 (48.8)		
	>40,000	50 (29.6)		111 (65.7)		8 (4.7)					169 (18.1)		
**Loot box**													
	Yes	54 (51.9)		45 (43.3)		5 (4.8)		.002			104 (11.2)		
	No	208 (35.3)		363 (61.6)		18 (3.1)					589 (63.2)		
	Missing	95 (39.7)		133 (55.6)		11 (4.6)					239 (25.6)		
**Time at home**													
	Much more	269 (47.4)		284 (50.0)		15 (2.6)		<.001			568 (60.9)		
	Slightly more	71 (28.2)		168 (66.7)		13 (5.2)					252 (27.0)		
	Unchanged	15 (14.0)		86 (80.4)		6 (5.6)					107 (11.5)		
	Less time	2 (40.0)		3 (60.0)		0 (0.0)					5 (0.5)		
**Change in alcohol habits**													
	More alcohol	49 (50.5)		43 (44.3)		5 (5.2)		<.001			97 (10.4)		
	Unchanged	133 (27.7)		339 (70.6)		8 (1.7)					480 (51.5)		
	Less alcohol	117 (51.1)		98 (42.8)		14 (6.1)					229 (24.6)		
	Does not drink	58 (46.0)		61 (48.4)		7 (5.6)					126 (13.5)		
**Change in exercise habits**													
	More exercise	87 (35.5)		147 (60.0)		11 (4.5)		<.001			245 (26.3)		
	Unchanged	89 (29.1)		208 (68.0)		9 (2.9)					306 (32.8)		
	Less exercise	168 (49.3)		161 (47.2)		12 (3.5)					341 (36.6)		
	Never	13 (32.5)		25 (62.5)		2 (5.0)					40 (4.3)		
**In school**													
	Yes	107 (59.4)		66 (36.7)		7 (3.9)		<.001			180 (19.3)		
	No	250 (33.2)		475 (63.2)		27 (3.6)					752 (80.7)		
**School performance**													
	Unchanged	26 (47.3)		28 (50.9)		1 (1.8)		.08			55 (5.9)		
	Better	26 (65.0)		11 (27.5)		3 (7.5)					40 (4.3)		
	Worse	55 (64.7)		27 (31.8)		3 (3.5)					85 (9.1)		
	Missing	250 (33.2)		475 (63.2)		27 (3.6)					752 (80.7)		
**School attendance**													
	Unchanged	61 (57.0)		44 (41.1)		2 (1.9)		<.001			107 (11.5)		
	Less	25 (69.4)		11 (30.6)		0 (0.0)					36 (3.9)		
	More	21 (56.8)		11 (29.7)		5 (13.5)					37 (4.0)		
	Missing	250 (33.2)		475 (63.2)		27 (3.6)					752 (80.7)		
**PGS-I^c^**													
	No problem with gambling	157 (32.6)		310 (64.3)		15 (3.1)		<.001			482 (51.7)		
	Low risk of gambling problems	40 (43.0)		48 (51.6)		5 (5.4)					93 (10.0)		
	Moderate risk of gambling problems	39 (52.7)		34 (45.9)		1 (1.4)					74 (7.9)		
	Gambling problems	18 (56.3)		14 (43.8)		0 (0.0)					32 (3.4)		
	Missing	103 (41.0)		135 (53.8)		13 (5.2)					251 (26.9)		
**Kessler-6**													
	Score 0-4: no psychological distress	79 (20.8)		286 (75.3)		15 (3.9)		<.001			380 (40.8)		
	Score 5-24: moderate psychological distress	273 (50.5)		251 (46.4)		17 (3.1)					541 (58.0)		
	Missing	5 (45.5)		4 (36.4)		2 (18.2)					11 (1.2)		
**GASA^d^**													
	Addicted/problem gamer	40 (66.7)		17 (28.3)		3 (5.0)		<.001			60 (6.4)		
	Engaged gamer	36 (66.7)		17 (31.5)		1 (1.9)					54 (5.8)		
	No problem	281 (34.4)		507 (62.0)		30 (3.7)					818 (87.8)		

^a^N/A: not applicable.

^b^A currency exchange rate of SEK 1=US $0.11 is applicable.

^c^PGS-I: Problem Gambling Severity Index.

^d^GASA: Game Addiction Scale for Adolescents.

#### Ages of 16 Years to 24 Years

In this age group, we found significant differences in disposable income (*P*=.04), time spent at home (*P*=.002), and school attendance (*P*=.02; Table 3).

**Table 3:** Gaming behavior by demographic characteristics among participants 16 years to 24 years of age.

Characteristics			Gaming behavior, n (%)							*P* value			Participants, n (%)
			Increased		Unchanged		Decreased					Total	
Total sample			87 (56.9)		59 (38.6)		7 (4.6)		N/A^a^			153 (100)	
**Gender**													
	Female	34 (50.0)		31 (45.6)		3 (4.4)		.27			68 (44.4)		
	Male	53 (62.4)		28 (32.9)		4 (4.7)					85 (55.6)		
**Employment status**													
	Studying	59 (57.3)		38 (36.9)		6 (5.8)		.89			103 (67.3)		
	Employed	22 (56.4)		16 (41.0)		1 (2.6)					39 (25.5)		
	Unemployed	5 (62.5)		3 (37.5)		0 (0.0)					8 (5.2)		
	Retired	—^b^		—		—					—		
	Other	1 (33.3)		2 (66.7)		0 (0.0)					3 (2.0)		
**Level of education**													
	Primary school	27 (71.1)		10 (26.3)		1 (2.6)		.25			38 (24.8)		
	Secondary school	43 (53.8)		32 (40.0)		5 (6.3)					80 (52.3)		
	University	17 (48.6)		17 (48.6)		1 (2.9)					35 (22.9)		
	Other	—		—		—					—		
**Disposable income (SEK^c^)**													
	<20,000	64 (53.8)		48 (40.3)		7 (5.9)		.04			119 (77.8)		
	20,000-40,000	23 (74.2)		8 (25.8)		0 (0.0)					31 (20.3)		
	>40,000	0 (0.0)		3 (100)		0 (0.0)					3 (2.0)		
**Loot box**													
	Yes	16 (69.6)		6 (26.1)		1 (4.3)		.19			23 (15.0)		
	No	33 (49.3)		32 (47.8)		2 (3.0)					67 (43.8)		
	Missing	38 (60.3)		21 (33.3)		4 (6.3)					63 (41.2)		
**Time at home**													
	Much more	68 (64.8)		35 (33.3)		2 (1.9)		.002			105 (68.6)		
	Slightly more	18 (47.4)		17 (44.7)		3 (7.9)					38 (24.8)		
	Unchanged	0 (0.0)		7 (77.8)		2 (22.2)					9 (5.9)		
	Less time	1 (100)		0 (0.0)		0 (0.0)					1 (0.7)		
**Change in alcohol habits**													
	More alcohol	8 (42.1)		10 (52.6)		1 (5.3)		.14			19 (12.4)		
	Unchanged	24 (54.5)		20 (45.5)		0 (0.0)					44 (28.8)		
	Less alcohol	35 (57.4)		23 (37.7)		3 (4.9)					61 (39.9)		
	Does not drink	20 (69.0)		6 (20.7)		3 (10.3)					29 (19.0)		
**Change in exercise habits**													
	More exercise	30 (53.6)		25 (44.6)		1 (1.8)		.22			56 (36.6)		
	Unchanged	13 (43.3)		15 (50.0)		2 (6.7)					30 (19.6)		
	Less exercise	40 (67.8)		16 (27.1)		3 (5.1)					59 (38.6)		
	Never	4 (50.0)		3 (37.5)		1 (12.5)					8 (5.2)		
**In school**													
	Yes	58 (56.9)		39 (38.2)		5 (4.9)		.96			102 (66.7)		
	No	29 (56.9)		20 (39.2)		2 (3.9)					51 (33.3)		
**School performance**													
	Unchanged	9 (36.0)		15 (60.0)		1 (4.0)		.15			25 (16.3)		
	Better	13 (61.9)		7 (33.3)		1 (4.8)					21 (13.7)		
	Worse	36 (64.3)		17 (30.4)		3 (5.4)					56 (36.6)		
	Missing	29 (56.9)		20 (39.2)		2 (3.9)					51 (33.3)		
**School attendance**													
	Unchanged	30 (53.6)		25 (44.6)		1 (1.8)		.02			56 (36.6)		
	Less	16 (66.7)		8 (33.3)		0 (0.0)					24 (15.7)		
	More	12 (54.5)		6 (27.3)		4 (18.2)					22 (14.4)		
	Missing	29 (56.9)		20 (39.2)		2 (3.9)					51 (33.3)		
**PGS-I^d^**													
	No problem with gambling	24 (53.3)		20 (44.4)		1 (2.2)		.76			45 (29.4)		
	Low risk of gambling problems	7 (43.8)		8 (50.0)		1 (6.3)					16 (10.5)		
	Moderate risk of gambling problems	13 (65.0)		7 (35.0)		0 (0.0)					20 (13.1)		
	Gambling problems	2 (40.0)		3 (60.0)		0 (0.0)					5 (3.3)		
	Missing	41 (61.2)		21 (31.3)		5 (7.5)					67 (43.8)		
**Kessler-6**													
	Score 0-4: no psychological distress	10 (40.0)		14 (56.0)		1 (4.0)		.16			25 (16.3)		
	Score 5-24: moderate psychological distress	74 (60.7)		44 (36.1)		4 (3.3)					122 (79.7)		
	Missing	3 (50.0)		1 (16.7)		2 (33.3)					6 (3.9)		
**GASA^e^**													
	Addicted/problem gamer	13 (76.5)		3 (17.6)		1 (5.9)		.20			17 (11.1)		
	Engaged gamer	9 (75.0)		3 (25.0)		0 (0.0)					12 (7.8)		
	No problem	65 (52.4)		53 (42.7)		6 (4.8)					124 (81.0)		

^a^N/A: not applicable.

^b^No responses to this category.

^c^A currency exchange rate of SEK 1=US $0.11 is applicable.

^d^PGS-I: Problem Gambling Severity Index.

^e^GASA: Game Addiction Scale for Adolescents.

#### Ages of 25 Years to 39 Years

In this age group, we found significant differences in employment status (*P*=.004), disposable income (*P*=.01), time spent at home (*P*=.005), changes in alcohol consumption (*P*=.003), changes in exercise habits (*P*=.001), whether the respondents were studying (*P*=.007), and Kessler score (*P*<.001; Table 4).

**Table 4:** Gaming behavior by demographic characteristics among participants 25 years to 39 years of age.

Characteristics			Gaming behavior, n (%)							*P* value			Participants, n (%)
			Increased		Unchanged		Decreased						
Total sample			146 (50.9)		126 (43.9)		15 (5.2)		N/A^a^			287 (100)	
**Gender**													
	Female	81 (53.3)		65 (42.8)		6 (3.9)		.48			152 (53.0)		
	Male	65 (48.1)		61 (45.2)		9 (6.7)					135 (47.0)		
**Employment status**													
	Studying	33 (76.7)		9 (20.9)		1 (2.3)		.004			43 (15.0)		
	Employed	93 (44.3)		104 (49.5)		13 (6.2)					210 (73.2)		
	Unemployed	15 (68.2)		7 (31.8)		0 (0.0)					22 (7.7)		
	Retired	—^b^		—		—					—		
	Other	5 (41.7)		6 (50.0)		1 (8.3)					12 (4.2)		
**Level of education**													
	Primary school	5 (50.0)		5 (50.0)		0 (0.0)		.74			10 (3.5)		
	Secondary school	53 (46.1)		54 (47.0)		8 (7.0)					115 (40.0)		
	University	80 (54.1)		61 (41.2)		7 (4.7)					148 (51.6)		
	Other	8 (57.1)		6 (42.9)		0 (0.0)					14 (4.9)		
**Disposable income (SEK^c^)**													
	<20,000	52 (66.7)		21 (26.9)		5 (6.4)		.01			78 (27.2)		
	20,000-40,000	82 (45.3)		91 (50.3)		8 (4.4)					181 (63.1)		
	>40,000	12 (42.9)		14 (50.0)		2 (7.1)					28 (9.8)		
**Loot box**													
	Yes	28 (58.3)		17 (35.4)		3 (6.3)		.45			48 (16.7)		
	No	84 (49.7)		77 (45.6)		8 (4.7)					169 (58.9)		
	Missing	34 (48.6)		32 (45.7)		4 (5.7)					70 (24.4)		
**Time at home**													
	Much more	105 (59.3)		65 (36.7)		7 (4.0)		.005			177 (61.7)		
	Slightly more	33 (38.8)		45 (52.9)		7 (8.2)					85 (29.6)		
	Unchanged	8 (32.0)		16 (64.0)		1 (4.0)					25 (8.7)		
	Less time	—		—		—					—		
**Change in alcohol habits**													
	More alcohol	20 (62.5)		9 (28.1)		3 (9.4)		.003			32 (11.1)		
	Unchanged	53 (41.4)		73 (57.0)		2 (1.6)					128 (44.6)		
	Less alcohol	49 (58.3)		28 (33.3)		7 (8.3)					84 (29.2)		
	Does not drink	24 (55.8)		16 (37.2)		3 (7.0)					43 (15.0)		
**Change in exercise habits**													
	More exercise	32 (47.8)		29 (43.3)		6 (9.0)		.001			67 (23.3)		
	Unchanged	34 (37.4)		53 (58.2)		4 (4.4)					91 (31.7)		
	Less exercise	77 (65.3)		37 (31.4)		4 (3.4)					118 (41.1)		
	Never	3 (27.3)		7 (63.6)		1 (9.1)					11 (3.8)		
**In school**													
	Yes	43 (68.3)		18 (28.6)		2 (3.2)		.007			63 (22.0)		
	No	103 (46.0)		108 (48.2)		13 (5.8)					224 (78.0)		
**School performance**													
	Unchanged	15 (65.2)		8 (34.8)		0 (0.0)		.13			23 (8.0)		
	Better	10 (66.7)		3 (20.0)		2 (13.3)					15 (5.2)		
	Worse	18 (72.0)		7 (28.0)		0 (0.0)					25 (8.7)		
	Missing	103 (46.0)		108 (48.2)		13 (5.8)					224 (78.0)		
**School attendance**													
	Unchanged	26 (66.7)		12 (30.8)		1 (2.6)		.80			39 (13.6)		
	Less	8 (80.0)		2 (20.0)		0 (0.0)					10 (3.5)		
	More	9 (64.3)		4 (28.6)		1 (7.1)					14 (4.9)		
	Missing	103 (46.0)		108 (48.2)		13 (5.8)					224 (78.0)		
**PGS-I^d^**													
	No problem with gambling	55 (45.8)		59 (49.2)		6 (5.0)		.48			120 (41.8)		
	Low risk of gambling problems	23 (56.1)		15 (36.6)		3 (7.3)					41 (14.3)		
	Moderate risk of gambling problems	18 (58.1)		12 (38.7)		1 (3.2)					31 (10.8)		
	Gambling problems	12 (66.7)		6 (33.3)		0 (0.0)					18 (6.3)		
	Missing	38 (49.4)		34 (44.2)		5 (6.5)					77 (26.8)		
**Kessler-6**													
	Score 0-4: no psychological distress	21 (29.6)		43 (60.6)		7 (9.9)		<.001			71 (24.7)		
	Score 5-24: moderate psychological distress	124 (57.7)		83 (38.6)		8 (3.7)					215 (74.9)		
	Missing	1 (100)		0 (0.0)		0 (0.0)					1 (0.3)		
**GASA^e^**													
	Addicted/problem gamer	21 (65.6)		9 (28.1)		2 (6.3)		.14			32 (11.1)		
	Engaged gamer	16 (66.7)		7 (29.2)		1 (4.2)					24 (8.4)		
	No problem	109 (47.2)		110 (47.6)		12 (5.2)					231 (80.5)		

^a^N/A: not applicable.

^b^No responses to this category.

^c^A currency exchange rate of SEK 1=US $0.11 is applicable.

^d^PGS-I: Problem Gambling Severity Index.

^e^GASA: Game Addiction Scale for Adolescents.

### Comparison of Increased Gaming in Different Outcomes (Unweighted Data)

The multivariable analysis using binary logistic regression models of the potential predictors of increased changes (yes versus no) are presented in the following sections.

#### All Age Groups

Increased gaming was significantly negatively correlated with the age group of 40 years to 59 years (OR 0.43, 95% CI 0.27-0.68) and ≥60 years (OR 0.57, 95% CI 0.33-0.97) as well as with much more time spent at home (OR 3.96, 95% CI 2.15-7.28). We also found a significant correlation with drinking less alcohol (OR 1.93, 95% CI 1.34-7.28) and self-reported not drinking alcohol (OR 1.66, 95% CI 1.05-2.61). Increased gaming was also significantly correlated with a Kessler score greater than 5 (OR 2.44, 95% CI 1.73-3.44) and with the GASA categories of engaged gamer (OR 2.27, 95% CI 1.23-4.20) and addicted/problem gamer (OR 2.37, 95% CI 1.26-4.47; Table 5).

**Table 5:** The likelihood of increased gaming behavior (increased vs unchanged/decreased) among all participants (n=932).

Characteristics		OR^a^ (95% CI)
**Age groups (years)**		
	16-24	Reference
	25-39	0.97 (0.63-1.50)
	40-59	0.43 (0.27-0.68)
	≥60	0.57 (0.33-0.97)
**Time at home**		
	Unchanged	Reference
	Much more	3.96 (2.15-7.28)
	Slightly more	1.72 (0.89-3.31)
	Less time	3.54 (0.44-28.28)
**Change in alcohol habits**		
	Unchanged	Reference
	More alcohol	1.62 (0.99-2.67)
	Less alcohol	1.93 (1.34-2.78)
	Does not drink	1.66 (1.05-2.61)
**Kessler-6**		
	Score 0-4: no psychological distress	Reference
	Score 5-24: moderate psychological distress	2.44 (1.73-3.44)
**GASA^b^**		
	No problem	Reference
	Engaged gamer	2.27 (1.23-4.20)
	Addicted/problem gamer	2.37 (1.26-4.47)

^a^OR: odds ratio.

^b^GASA: Game Addiction Scale for Adolescents.

#### Ages of 16 Years to 24 Years

In this age group, no correlations were found (Table 6).

**Table 6:** The likelihood of increased gaming behavior (increased vs unchanged/decreased) among participants 16 years to 24 years of age

Characteristics		OR^a^ (95% CI)
**Disposable income (SEK^b^)**		
	<20,000	Reference
	20,000-40,000	5.69 (0.5-65.0)
	>40,000	—^c^
**Loot box**		
	Yes	Reference
	No	0.66 (0.15-2.89)
**Time at home**		
	Unchanged	Reference
	Much more	—
	Slightly more	—
	Less time	—
**School attendance**		
	Unchanged	Reference
	Less	2.5 (0.61-10.3)
	More	1.39 (0.33-5.85)

^a^OR: odds ratio.

^b^A currency exchange rate of SEK 1=US $0.11 is applicable.

^c^Could not be estimated.

#### Ages of 25 Years to 39 Years

In this age group, employment was negatively correlated with a self-reported increase in gaming (OR 0.41, 95% CI 0.18-0.92). Self-reporting less exercise was positively correlated with increased gaming (OR 2.27, 95% CI 1.20-4.27), and Kessler scores greater than 5 were positively correlated with a self-reported increase in gaming activity (OR 2.36, 95% CI 1.27-4.41; Table 7).

**Table 7:** The likelihood of increased gaming behavior (increased vs unchanged/decreased) among participants 25 years to 39 years of age.

Characteristics			OR^a^ (95% CI)
**Employment status**			
	Studying	Reference	
	Employed	0.41 (0.18-0.92)	
	Unemployed	0.82 (0.24-2.74)	
	Retired	—^b^	
	Other	0.30 (0.07-1.30)	
**Change in alcohol habits**			
	Unchanged	Reference	
	More alcohol	1.49 (0.62-3.58)	
	Less alcohol	1.61 (0.88-2.97)	
	Does not drink	1.65 (0.77-3.55)	
**Change in exercise habits**			
	Unchanged	Reference	
	More	1.29 (0.64-2.59)	
	Less	2.27 (1.20-4.27)	
	Never	0.60 (0.14-2.60)	
**Time at home**			
	Unchanged	Reference	
	Much more	1.60 (0.59-4.32)	
	Slightly more	0.94 (0.34-2.64)	
	Less time	—	
**Kessler-6**			
	Score 0-4: no psychological distress	Reference	
	Score 5-24: moderate psychological distress	2.36 (1.27-4.41)	

^a^OR: odds ratio.

^b^Could not be estimated.

## Discussion

This cross-sectional study aimed to look at self-reported changes in gaming behavior during the third wave of the COVID-19 pandemic in Sweden. We also wanted to look at potential risk factors for problematic gaming during the pandemic, including gaming patterns, gambling behavior, psychological distress, a number of sociodemographic characteristics, health factors, and school situation during the pandemic. We used data from a web panel of 1501 respondents who answered questions on gaming and gambling. The results on gambling are presented elsewhere.

### Principal Findings

We found several factors associated with changed gaming behavior, but on further analysis, only psychological distress (all age groups analyzed together and the 25–39-year-old age group), drinking less alcohol (all age groups analyzed together), spending more time at home (all age groups analyzed together), gaming problems (all age groups analyzed together), and exercising less (25–39-year-old age group) were positively correlated with a self-reported increase in gaming activity. Being employed (25–39-year-old age group) and being over 40 years of age (all age groups analyzed together) were negatively correlated with increased gaming. We did not find any significant correlations in the 16–24-year-old age group.

### Comparison With Prior Work

Research from the early phases of the COVID-19 pandemic showed worrying figures for increased screen time among young people, raising questions about whether this would continue and how it would affect the younger population [55-57]. Gaming disorder has been recognized as a public health problem of importance, but the majority of people who engage in gaming do not fulfil the criteria for gaming disorder [58]. In most studies, the overall prevalence of gaming disorder is around 3% [48]. In our study, 62% of respondents self-reported that they sometimes played video games. We found 38% self-reported an increase in gaming since the start of the COVID-19 pandemic. When looking at gaming and possible changes in the younger population, in the 16–24-year-old age group, 57% self-reported an increase in gaming, and in the 25–39-year-old age group, 51% reported an increase in gaming. Other researchers have seen similar results. Frequencies and screen time had increased during the COVID-19 pandemic when Paschke et al [59] looked at those aged 10 years to 17 years and compared their usage frequency and screen time from before the pandemic with their behavior in lockdown. Lemenager et al [60] found the same tendency: 71.4% of participants estimated a general increase in their online media consumption during the lockdown, and some 10% self-reported a rise in gaming activity during the COVID-19 pandemic, of whom men aged between 18 years and 24 years showed the highest increase in gaming. Studies from before the COVID-19 pandemic had shown the same tendency, with young men reported to play computer games more frequently and for longer duration [61,62], making them vulnerable to developing addictive gaming behaviors [63-65]. Balhara et al [58] looked at university students’ gaming behavior during the COVID-19 pandemic and found those who used gaming as a tool to reduce stress showed an increase in gaming activity during the pandemic. Before the COVID-19 pandemic, it was known that gamers who gave escapism (a coping technique to handle negative emotions) as a reason for gaming were more commonly problem gamers [66,67].

When analyzing the whole sample who reported increased gaming, we found positive associations with engaged gaming but also with addicted/problem gaming; this relationship was not seen in the 2 younger groups. Increased time spent gaming is a risk factor for developing gaming problems/addiction [63-65]. Previous studies have shown a huge male predominance in problematic gaming behavior [27,68,69]. Men have been reported to play computer games more frequently and for longer duration [61,62], whereas there is a female predominance in smartphone use [70]. Massively multiplayer online role-playing games (MMORPGs) and multiplayer online battle arenas (MOBAs), both with a predominance of male players, have been found to have an addictive potential due to their specific structural characteristics of advancement and social interactions [28,29,71-74]. Researchers have also looked at the cortical region and found that gender differences in IGD might be associated with different cortical thickness in and around the posterior cingulate cortex, the region thought to be involved in cognitive control and reward/loss processing and hence thought to play a role in addiction [68]. These are plausible explanations for the gender differences. In this study, we did not see that gender was associated with self-reported increased gaming, possibly because the numbers were too small.

During the COVID-19 pandemic, the media, schools, and parents all wondered whether remote learning would see schoolchildren spending more time using digital media, not including homework. This was to some extent true for April 2020 to June 2020, when a 69%-76% rise in online media use was reported for children in the United Kingdom, Spain, and Belgium compared with a 36% rise in the Swedish population [56,57]. Although not immediately comparable, the difference can to some extent be explained by the fact that, in the Swedish material, the majority of pupils were aged 13 years to 16 years, compared with an age range of 5 years to 12 years in the other countries, and while the Swedish children were online for the whole school day, the children in the other countries did their schoolwork under their parents’ supervision [56,57].

In India, which has seen intermittent total lockdowns throughout the pandemic, meaning people have had to stay at home, an increase in gaming has been seen in those aged 25 years to 35 years [14]. There have also been public health efforts by the WHO to encourage people to engage in gaming (#PlayApartTogether) to promote social distancing and prevent the virus spread [21]. In our study, looking at all age groups together, we saw a correlation between staying home much more and self-reporting an increase in gaming. The relationship was not found in the 2 age groups under 40 years of age. It is important to bear in mind that our sample was rather limited in size, and possibly a larger sample size would show additional correlations.

Being 40 years old or more seemed to reduce the reporting of increased gaming, not surprisingly since gaming is more common in younger people [61,62]. We found a positive correlation between less exercise and increased gaming in the age group of 25 years to 39 years. Sedentary lifestyles and increased gaming have been known to co-occur during the COVID-19 pandemic, making researchers call for parents, schools, and decision makers to mandate physical activity and keep outdoor facilities open as long as possible, even in lockdowns [55,75]. Psychological distress was positively associated with increased gaming in the whole sample as well as in those aged 25 years to 39 years. The association of excessive gaming with comorbidities such as sleeping disorders, obesity, depression, and anxiety is well known [68,76-78], marking out the group who self-reported increased gaming *and* psychological distress as vulnerable and a focus of concern. The same was observed by King et al [18]: Some individuals may develop an increasingly unhealthy pattern of gaming due to pandemic-related psychological distress, because they find gaming relieves stress. We found respondents aged 25 years to 39 years who reported increased gaming during the pandemic were less likely to be in work. Unemployment might facilitate their gaming activities, enabling them to spend more time at home, possibly under greater stress.

When examining those aged 16 years to 24 years who reported increased gaming, we did not find any correlations with associated factors. One possible reason why those in this age group who reported increased gaming did not seem to suffer from psychological distress could be that gaming involves social aspects and thus increased their ability to cope with social isolation in a functional way. This would seem to be confirmed by a report by Bora [14] showing higher usage in multiplayer modes. Playing video games together helped people reduce feelings of loneliness and stress and to stay in touch with friends [58].

Gaming was already viewed by some in a positive light for helping to develop cognitive skills such as reasoning, spatial awareness, and problem-solving [74], and it is now also considered a way of maintaining social contact during lockdown. It would be desirable for authorities to issue recommendations for preferred types of video games that enhance social activity and physical health while avoiding the pitfalls of unhealthy gaming.

### Limitations

This study has several limitations. The study was based on a self-assessment questionnaire, rather than a standardized, structured clinical interview that would allow a more accurate assessment. Against that, questionnaires are widely used in epidemiological studies, and in other studies, they have been considered to give a satisfactory picture of the situation [39,41]. Our data were based on a web panel survey, and although the study sample was designed and weighted to represent the general population, it is hard to know whether the respondents’ original choice to enroll in a web panel is associated with other characteristics and, in this case, with gaming habits that differ from those of their peers in the general population. Our sample size is too small to draw generalized conclusions.

### Strengths

Despite the extreme interest in the possible increase in gaming during the COVID-19 pandemic, whether in popular science or more clinical and scientific contexts, there is still a significant lack of studies focusing on the younger population. This study contributes important information about possible changes in gaming behavior during the COVID-19 pandemic.

### Conclusion

Those who reported increased gaming during the COVID-19 pandemic were more likely to be 16 years to 39 years old. In those aged 16 years to 24 years, increased gaming was not associated with any risk factors. In the 25–39-year-old age group, the increase was associated with psychological distress, reporting less exercise, and being unemployed. COVID-19 may present a risk factor for increased online gaming in a small but vulnerable group. More research and preferably longitudinal studies are needed in the field of gaming and the effects of the COVID-19 pandemic.

## Abbreviations

DSM-5: *Diagnostic and Statistical Manual of Mental Disorders*
GASA: game addiction scale for adolescents
ICC: International Chamber of Commerce
ICD: International Classification of Diseases
IGD: internet gaming disorder
MMORPG: massive multiplayer online role-playing game
MOBA: multiplayer online battle arena
OR: odds ratio
PGS-I: Problem Gambling Severity Index
WHO: World Health Organization
